# Improvement of Estrogen Deficiency Symptoms by the Intake of Long-Term Fermented Soybeans (Doenjang) Rich in *Bacillus* Species through Modulating Gut Microbiota in Estrogen-Deficient Rats

**DOI:** 10.3390/foods12061143

**Published:** 2023-03-08

**Authors:** Ting Zhang, Yu Yue, Su-Ji Jeong, Myeong-Seon Ryu, Xuangao Wu, Hee-Jong Yang, Chen Li, Do-Youn Jeong, Sunmin Park

**Affiliations:** 1 Department of Bioconvergence, Hoseo University, 20 hoseoro79bun-gil, Baebang-yup, Asan 31499, Republic of Korea; 2 Obesity/Diabetes Research Center, Department of Food and Nutrition, Hoseo University, Asan 31499, Republic of Korea; 3 Sunchang Research Center for Fermentation Microbes, Department of R & D, Microbial Institute for Fermentation Industry, 61-27 Minsokmaeul-gil, Sunchang-Gun 56048, Republic of Korea

**Keywords:** long-term fermented soybeans, estrogen deficiency, *Bacillus*, biogenic amines, gut microbiota, metagenome function

## Abstract

Traditionally made doenjang (TMD) produced by the long-term fermentation of soybeans with salt may improve symptoms of estrogen deficiency. We aimed to evaluate the effects of four TMD types, containing low and high amounts of *Bacillus* species and biogenic amines (HBHA, HBLA, LBHA, and LBLA), on energy, glucose, and lipid metabolism, by altering the gut microbiota in estrogen-deficient ovariectomized (OVX) rats. Their mechanisms were also examined. The OVX rats were divided into the control, cooked soybean (CSB), HBHA, LBHA, HBLA, and LBLA groups. Sham-operated rats were the normal control group. Serum 17β-estradiol concentrations were similar among all OVX groups. Tail skin temperatures, which are indicative of hot flashes, were higher in the control than the HBHA and HBLA groups and were similar to the normal control group. Weight gain and visceral fat mass were lower in the TMD and CSB intake groups but not as low as in the normal control group. Lean body mass showed a trend opposite to that of visceral fat in the respective groups. The hepatic triglyceride content decreased with the TMD intake compared to the control and CSB groups. mRNA expressions of the peroxisome proliferator-activated receptor-γ (*PPAR-γ*) and carnitine palmitoyltransferase-1 in the TMD and CSB groups were as high as in the normal control group, and the *PPAR-γ* mRNA expression was more elevated in the HBLA group than in the normal control group. The morphology of the intestines improved in the TMD groups compared to the control, and the HBHA and HBLA groups showed an enhanced improvement compared to the CSB group. The HBHA, HBLA, and LBHA groups increased the α-diversity of the cecal microbiota compared to the control. *Akkermenia* and *Lactobacillus* were higher in the HBLA and LBLA groups compared to the control. The expression of the estrogen, forkhead box proteins of the class-O subgroup, and insulin-signaling pathways were lower in the control group, and HBHA and HBLA prevented their decrement. In conclusion, long-term treatment with TMD containing high amounts of *Bacillus* potentially improves estrogen deficiency symptoms more than unfermented soybeans.

## 1. Introduction

Globally, life expectancy has increased by more than six years, according to the most current data released by the World Health Organization (WHO) [1]. The mean menopausal age is 52 and 50 years in European and Asian women, respectively, and women live for 30 years after menopause [2]. Although women have much higher levels of body fat than men, they have a lower premenopausal risk of metabolic diseases [3]. However, estrogen deficiency markedly disturbs energy, glucose, lipid, and bone metabolism to induce abdominal obesity, hyperglycemia, hypertension, dyslipidemia, and osteoporosis [3]. Hormone replacement therapy (HRT) offers protection against metabolic diseases but it has some adverse effects [4]. Thus, there is a constant effort to find alternative therapies for managing menopause and its related effects.

Soybeans contain high concentrations of isoflavonoids that act as phytoestrogens by mimicking estrogen in the body through their action on the estrogen receptors [5]. Soybean intake has been reported to alleviate menopausal symptoms, but the effects vary individually. A factor influencing the response to soybean intake is the gut microbiota composition of individuals [6,7]. Over 50% of isoflavonoids are glycated forms, such as daidzin, genistin, and glycitin. The glucose moiety is removed during digestion to produce the aglycone forms, daidzein, genistein, and glycitein [8]. Isoflavonoid aglycones have better phytoestrogen activity than glycated isoflavonoids. Equol, which is produced by intestinal bacteria from daidzein, is an isoflavone-derived metabolite with high estrogenic and antioxidant activity [9]. Fermented soybeans contain more isoflavonoid aglycones, thus producing more equol than unfermented soybeans [10].

Traditionally made doenjang (TMD) has been commercialized, and its quality is regulated by the Korean Food and Drug Administration to prevent harmful contamination. However, TMD contains different bacterial compositions and bioactive compounds such as isoflavonoids and biogenic amines. Over 20 TMD types have been collected to measure the bacterial composition and biogenic amines, and they are categorized into four types according to Bacillus and the biogenic amine contents. Each commercialized TMD has consistent bacterial and bioactive compositions, although, different ones have been shown to have different compositions and health benefits. Studying the differential effects of TMD on energy, glucose, and lipid metabolisms is imperative. However, no studies have determined the health benefits of different TMD types. It has been demonstrated earlier that *Bacillus* spp. in traditionally made kanjang, the salty water component produced after soybean fermentation, can improve estrogen deficiency symptoms in ovariectomized (OVX) rats. The intake of traditionally made kanjang with high *Bacillus* species (spp.) but not biogenic amine contents affects the gut microbiota composition to improve energy, fatty acid biosynthesis, and bile acid metabolism in estrogen-deficient rats [11]. Therefore, the bacteria and biogenic amines contained in TMD could also affect estrogen deficiency symptoms by altering the isoflavonoid content and gut microbiota composition.

However, no studies show how TMD intake may alleviate menopausal symptoms, including hot flashes, obesity, hyperglycemia, and dyslipidemia. The present study aimed to determine the levels of biogenic amines and *Bacillus* spp. in different TMD samples and reveal how they influence the disturbance of energy, glucose, and lipid metabolism differently by altering gut microbiota in estrogen-deficient rats generated with ovariectomy (OVX). The novel results show that different contents of *Bacillus* and biogenic amines modulate estrogen deficiency symptoms, including hot flashes and energy, lipid, and glucose disturbance, ands are related to improving insulin and estrogen signaling of the metagenome function in the gut microbiota of OVX rats. Furthermore, TMD with high *Bacillus* prevented estrogen deficiency symptoms the most, and biogenic amine contents in TMD did not influence the metabolic improvement in OVX rats.

## 2. Materials and Methods

### 2.1. TMD Production Process and Sample Collection

Five different batches of TMD samples from different provinces in Korea were collected. Five or more samples from each TMD batch were collected and assayed for bacteria counts and bioactive compounds. TMD in Korea is generally made in a two-stage process: First, meju is prepared by crushing boiled soybeans and then fermenting them in rice straw at about 20–25 °C for about 40–50 days. The second stage is fermentation in 16–18 Brix salt water for 40–60 days, and the solution is separated with the liquid fraction being kanjang and the solid fraction being TMD. A preliminary study measured sodium, beneficial and harmful bacteria, and the biogenic amine contents in TMD. Based on these data, four TMD products were selected for the in vivo trial to evaluate their effectiveness in alleviating menopausal symptoms. The selection criteria were based on the levels of beneficial bacteria and biogenic amines, as follows: (1) high beneficial bacteria plus low biogenic amines (HBLA), (2) high beneficial bacteria plus high biogenic amines (HBHA), (3) low beneficial bacteria plus low biogenic amines (LBLA), and (4) low beneficial bacteria plus high biogenic amines (LBHA).

The sodium content in the TMD was measured using inductively coupled plasma atomic emission spectroscopy (ICP-AES; Thermo IRIS Intrepid II XDL, Waltham, MA, USA) after proteins were digested with nitric acid according to the guidelines of the Korean Ministry of Food and Drug Safety (MFDS). The oxygen and acetylene flows were 10.00 L/min and 2.50 L/min, respectively. The flame type was air acetylene, and the wavelength was 589.0 nm. TMD was mixed with methanol and filtered to measure sodium contents in the TMD.

The isoflavonoid content in the filtrates was determined by high-performance liquid chromatography (HPLC) (Agilent 1200 series, Agilent Technologies, Santa Clara, CA, USA) equipped with a Shiseido UG 120 (4.6 × 250 mm, 5 μm, Osaka, Japan) column. The mobile phase was a mixture of acetonitrile and water (25:75, *v*/*v*; J.T. Baker, Philadelphia, PA, USA), the flow rate was 1.0 mL/min, the injection volume was 10 μL, and a fluorescence detector (FLD) was used (excitation: 360 nm, measurement 450 nm; Agilent Technologies, Santa Clara, CA, USA). The standards of isoflavonoids such as daidzein, daidzin, genistein, genistin, glycetein, and glycetin were purchased from Sigma-Aldrich (St. Louise, MO, USA).

The contents of the biogenic amines, such as histamine and tyramine, were determined as described previously [12]. TMD was mixed with an internal standard, 1,7-diaminoheptane (0.1 g/L, Sigma-Aldrich), and it was added into a saturated sodium carbonate (Na_2_CO_3_) (Sigma-Aldrich) solution and 1% dansyl chloride (Sigma-Aldrich) to make derivatives. Standards (histidine and tyramine) were prepared with 0.1–100 mg/L concentrations in a 0.01 N HCl solution. Ethyl ether (Samchun, Seoul, Republic of Korea) was mixed with the solution for 3 min, and the supernatants were separated and dissolved in acetonitrile (Duksan, Seoul, Republic of Korea). The biogenic amine contents were then measured by high-performance liquid chromatography (HPLC) analysis with a Cepcell Pak C18 column (2.0 × 250 mm) [12].

As mentioned below, the proportion of bacteria in TMD was calculated after carrying out bacterial measurement by the next-generation sequencing (NGS) method. The beneficial and pathogenic bacteria were designated according to the bacteria characteristics from previous studies [13,14].

### 2.2. Ovariectomy Procedure

Seventy female Sprague Dawley rats (aged 6 weeks, 167 ± 10 g) were purchased from DBL (Yeumsung-Kun, Republic of Korea) and were acclimated for one week in the animal facility at Hoseo University. The rats were housed in individual stainless-steel cages (23 °C, with a 12 h light/dark cycle). This study was conducted according to the Guide for the Care and Use of Laboratory Animals, funded by the National Institutes of Health (NIH) in the USA and with the approval of the Hoseo University Animal Care and Use Committee (2014-03).

The rats were anesthetized with subcutaneous injections of a ketamine/xylazine mixture (100 and 10 mg/kg body weight) during the ovariectomy (OVX) procedure [11]. After a mid-ventral incision, each ovary was removed by ligating the most proximal part of each oviduct, and both ovaries were dissected with scissors. The OVX groups included ten OVX rats per group, and ten rats had a sham operation.

### 2.3. Experimental Design

**Figure 1:**
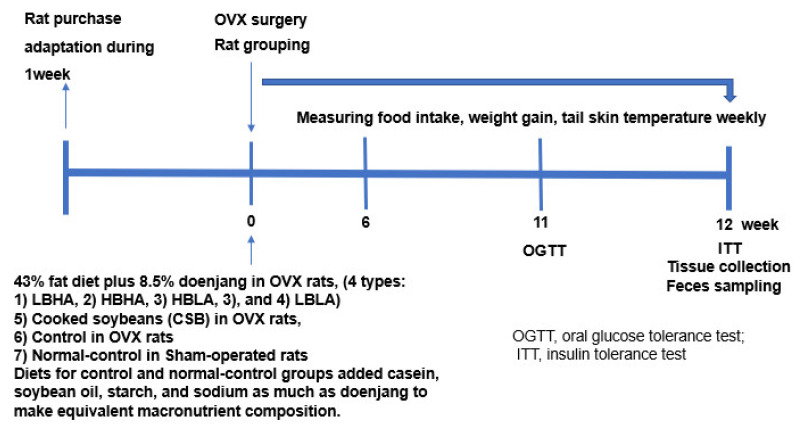
Experimental design of the animal study.

Figure 1 presents the experimental design. OVX rats were orally provided a diet including either 4 different types of lyophilized TMD according to Bacillus and biogenic amine contents to determine the different TMD effects on estrogen deficiency symptoms. Each group was provided with either 4 different TMDs, unfermented soybeans, or a diet containing no soybeans or TMD for the normal control group. TMD or soybeans were incorporated into the diet, and the control diet contained an equivalent nutrient composition to the TMD or soybean diets by adding nutrients to the soybeans.

Sixty OVX rats were randomly divided into groups as follows: (1) control, (2) lyophilized TMD with high *Bacillus* and low biogenic amines (HBLA), (3) high *Bacillus* and high biogenic amines (HBHA), (4) low *Bacillus* and low biogenic amines (LBLA), (5) low *Bacillus* bacteria and high biogenic amines (LBHA), and (6) cooked soybeans (CSB). Sham-operated rats were considered the normal control group and had a nutritionally equivalent diet as the other groups, but without soybean or TMD. During the 12 weeks of dietary intervention, the rats fasted overnight, and their food and water intake and body weight were measured at 10 am every Tuesday. At the end of the 12-week intervention, the rats were anesthetized with ketamine and xylazine, and blood was collected from the portal vein and the vena cava. The liver, abdominal fat, uterine, skeletal muscles in the legs, and feces in the cecum were dissected and stored in a −70 °C freezer.

### 2.4. Diet Preparation

All the groups were fed high-fat diets to exacerbate the menopausal symptoms in the OVX rats [15,16,17]. In a semi-purified method, the diet was generated based on an AIN 93 formulation for experimental animals with or without four different types of TMD or CSB. The primary sources of carbohydrates, proteins, and fats were starch plus sugar, casein, and lard (CJ Co., Seoul, Republic of Korea), respectively. The TMD or soybeans dosage was 8.5% in each diet according to the sodium contents designated in AIN 93 formulation. According to the nutrient composition of TMD, the contents of carbohydrates, protein, fat, and sodium were subtracted from diet composition to make their contents equivalent among all diets. When adding corresponding lyophilized TMD in HBHA, HBLA, LBHA, and LBLA, or CSB, the amounts of carbohydrates, protein, fat, and sodium in TMD or CSB were removed from starch, casein, soybean oil, and the mineral mixture from the corresponding group. The carbohydrate, protein, fat, and salt contents in each diet were 39.5, 17.1, 43.4 energy percent (EN%), and 5.9 g salt/kg diet, respectively. Each TMD powder was thoroughly blended into the vitamin and mineral mixture without sodium and sugar and sifted to remove lumps. The vitamin and mineral mixtures were mixed with the designated amounts of starch, casein, and lard and were then resifted. Each group was given a diet with an equivalent primary nutrient composition.

### 2.5. Tail Skin Temperature

Rat tail skin temperatures and surrogate measures of hot flashes in menopausal women were measured weekly during the sleep cycle using an infrared thermometer for small rodents (BIO-152-IRB, Bioseb, Chaville, France) three times and 10 min apart [17].

### 2.6. Fat and Skeletal Muscle Composition

After anesthetization, the rats were laid in a prone position with posterior legs with 90° flexion of the knee, hip, and ankle. Abdominal fat and lean body mass (LBM) were determined in the leg, abdomen, and hip upon the completion of scanning of the body using the dual-energy X-ray absorptiometer instrument (DEXA; Norland pDEXA Sabre; Norland Medical Systems Inc., Fort Atkinson, WI, USA) equipped with the appropriate software for the assessment in small animals [18].

### 2.7. Insulin Resistance and Lipid Profiles

The homeostasis model assessment estimate for assessing insulin resistance (HOMA-IR) was used to estimate insulin resistance according to the following equation:

HOMA_IR = fasting insulin (µIU/mL) × fasting glucose (mM)/22.5.

Serum glucose and insulin concentrations were measured using a Glucose Analyzer II (Beckman-Coulter, Palo Alto, CA, USA) and radioimmunoassay kits (Linco Research, Billerica, MA, USA). Serum 17β-estradiol levels were measured by ELISA (Enzo Life Sciences, Farmingdale, NY, USA). Serum lipid profiles were assayed using colorimetry kits for total cholesterol, HDL cholesterol (HDL-C), and triglycerides (Asan Pharmaceutical, Seoul, Republic of Korea). Serum LDL-C concentrations were calculated from the serum lipid concentrations using the Friedewald equation (LDL-C = total cholesterol − HDL-C − triglycerides/5). We determined serum tumor necrosis factor (TNF)-α and lipid peroxide (malondialdehyde) concentrations using the TNF-α ELISA kit (R & D Systems, Minneapolis, MN, USA) and lipid peroxide ELISA kit (Abcam, Cambridge, UK).

### 2.8. Gene Expression by the Real-Time PCR Method

Total RNA was extracted by mixing the liver pieces with phenol/guanidine isothiocyanate monophasic solution (TRIzol reagent; Gibco-BRL, Rockville, MD, USA) according to the manufacturer’s instructions. Equal amounts of the total RNA were used to synthesize cDNA using Superscript III reverse transcriptase. A polymerase chain reaction (PCR) was implemented using high-fidelity Taq DNA polymerase. cDNAs were equally added to the SYBR Green mix (Bio-Rad, Richmond, CA, USA) along with primers for specific genes using a real-time PCR instrument (Bio-Rad) under optimal conditions for thermal cycling. The expressions of the genes of interest were normalized to that of the β-actin gene. The mRNA expressions of peroxisome proliferator-activated receptor gamma (*PPAR-γ*), sterol regulatory element-binding protein 1 (*SREBP-1c*), and carnitine palmitoyltransferase I (*CPT-1*) were determined using corresponding primers, as described previously [19]. A cycle of threshold (CT) for each sample was assessed using a real-time PCR method. Expression levels of the islet genes were quantitated using the comparative CT method (ΔΔCT method).

### 2.9. Histology of the Large Intestines

After dissecting the large intestines, the rats were sequentially perfused with saline and a 4% paraformaldehyde solution (pH 7.2). The large intestinal tissues were immediately dissected and post-fixed with 4% paraformaldehyde overnight at room temperature [20]. Two serial 5 μm paraffin-embedded large intestine sections were randomly chosen, and they were stained with hematoxylin–eosin (H-E) and Alcian blue–perchloric acid (PAS). After staining, the area of the intestinal villi in the H-E stained sections was measured using a Zeiss Axiovert microscope (Jena, Germany) with the DIXI Imaging Solution at 10× magnification. The length and width of the villi, the height of the crypt, and the impaired cells were counted in the H-E section. The relative area of the impaired cells was scored 0–3 as 0 (none or minimal), 1 (mild), 2 (moderate), and 3 (severe). The percentage of intestinal goblet cells producing mucin, indicated by blue staining, was calculated using the Alcian blue–PAS-stained sections.

### 2.10. Serum Short-Chain Fatty Acids (SCFA) Concentrations and Gut Microbiome

Serum was separated from the portal vein blood and mixed with ethanol (Duksan, Republic of Korea). A total of 1N HCl was blended into the mixture (100:1) and centrifuged at 15,000 rpm, for 15 min, at 4 °C. SCFA concentrations in the supernatants were assayed using a gas chromatograph (Clarus 680 GAS, PerkinElmer, Waltham, MA, USA) equipped with an Elite-FFAP 30 m × 0.25 mm × 0.25 μm capillary column. Helium was used as the carrier gas at a flow rate of 1 mL/min [21]. Exogenous acetate, propionate, and butyrate (1 mM; Sigma Co., St. Louis, MO, USA) were used as the external standards.

Metagenome sequencing used the NGS procedures to investigate TMD and fecal microbiome communities from the cecum [16]. According to the manufacturer’s instructions, bacterial DNA was extracted from the cecal feces using a Power Water DNA Isolation Kit (Qiagen, Valencia, CA, USA). DNA was amplified with 16S amplicon primers by PCR, and libraries were prepared for PCR products according to the GS FLX plus library prep guide, as described previously [22]. According to the manufacturer’s instructions, the PCR amplification program was run with 16S universal primers in the FastStart High-Fidelity PCR System (Roche, Basel, Switzerland). The bacterial DNA of the cecal feces was sequenced using the Illumina MiSeq standard operating procedure and a Genome Sequencer FLX plus (454 Life Sciences) (Macrogen, Seoul, Republic of Korea).

The 16S amplicon sequences were processed using Mothur v.1.36 package. The Miseq standard operation procedure was used to identify cecal bacterial taxonomy, and bacterial counts were conducted on each fecal sample. Sequences were aligned using the Silva reference alignment v.12350, and bacteria counts and identifications for all taxa were determined as described previously [21,22]. Relative bacteria counts were calculated in the taxonomic assignment order for each sample. PCoA results for gut bacteria were visualized using the R package (Vienna, Austria).

### 2.11. Metabolic Functions of the Gut Microbiomes by PICRUSt2 Pipeline Analysis

Metabolic functions of gut microbiota were predicted from the FASTA files and count tables of fecal bacteria using the Phylogenetic Investigation of Communities by Reconstruction of Unobserved States 2 (PICRUSt2) software [23]. Metabolic functions were predicted using the Kyoto Encyclopedia of Genes and Genomes (KEGG) Orthologues (KO) and mapped by the KEGG mapper (https://www.genome.jp/kegg/tool/map_pathway1.html, 9 September 2022) [22]. The gut microbiome was used to explore the differences in the metabolic functions among the groups.

### 2.12. Statistical Analysis

SAS software version 7 (SAS Institute, Cary, NC, USA) was used for statistical analysis. The optimal sample size was evaluated using a G power program (power = 0.90 and effect size = 0.5), and the calculated sample size was 10 per group. Results are expressed as means ± standard deviations (SD) when the results were normally distributed as confirmed using the Proc univariate procedure. Measurements were statistically analyzed using one-way ANOVA. The significant differences among the groups were assessed using Tukey’s test, and differences were considered significant at *p* < 0.05.

## 3. Results

### 3.1. Characteristics of TMD according to Bacillus spp. and Biogenic Amine Concentrations

**Figure 2:**
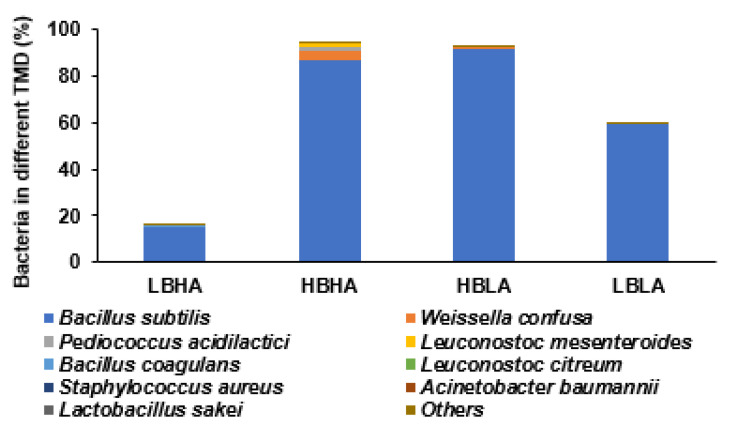
Bacterial composition at the genus level in traditionally made doenjang. Bacteria in five different traditionally made doenjang (TMD) samples were purchased, and bacteria contents were measured using the next-generation sequencing (NGS) method. The bacteria compositions were exhibited at the species level, and the contents of bacteria less than 0.01% were summed and presented as the others. Since the bacteria compositions of TMD samples influenced TMD function, they could be used for their quality control. Bars represent means (*n* = 5).

TMD was fermented with about 4–5% salts, and the predominant bacteria in the TMD was the *Bacillus* spp. (Table 1). However, the bacterial compositions were disparate among different TMD samples due to the varying average temperatures during the year and the salt content. The amounts of biogenic amines and sodium were different according to the bacterial compositions. We chose four different doenjang varieties containing biogenic amines and *Bacillus* content. The water content of the four TMD products was about 50% (50–59%). Two TMD products were rich in *Bacillus* spp. (HB) and contained high amounts of biogenic amines (histamines and tyramine; HA) (Table 1). The bacterial compositions of the TMD samples measured by NGS are present in Figure 2. The bacterial contents varied among different TMD samples, and they were lower in the order of LBHA, HBHA, HBLA, and LBLA. The differences might be linked to the environmental conditions when making doenjang. Furthermore, HBHA and HBLA contained 95% beneficial bacteria, primarily *Bacillus* spp. The bacteria contents in the CSB were not included since it was not expected to contain bacteria after boiling soybeans.

### 3.2. Isoflavonoid Contents

LBHA, HBHA, and HBLA contained isoflavonoid aglycones such as daidzein, genistein, and glycitein but not glycated isoflavonoids such as daidzin, genistin, and glycitin. The results indicated that glycated isoflavonoids were converted into isoflavonoid aglycones (Table 1). However, LBLA contained fewer total isoflavonoids and isoflavonoid aglycones than the other TMD samples (Table 1).

### 3.3. Uterine Weight, Serum 17β-Estradiol Levels, and Tail Skin Temperature

**Figure 3:**
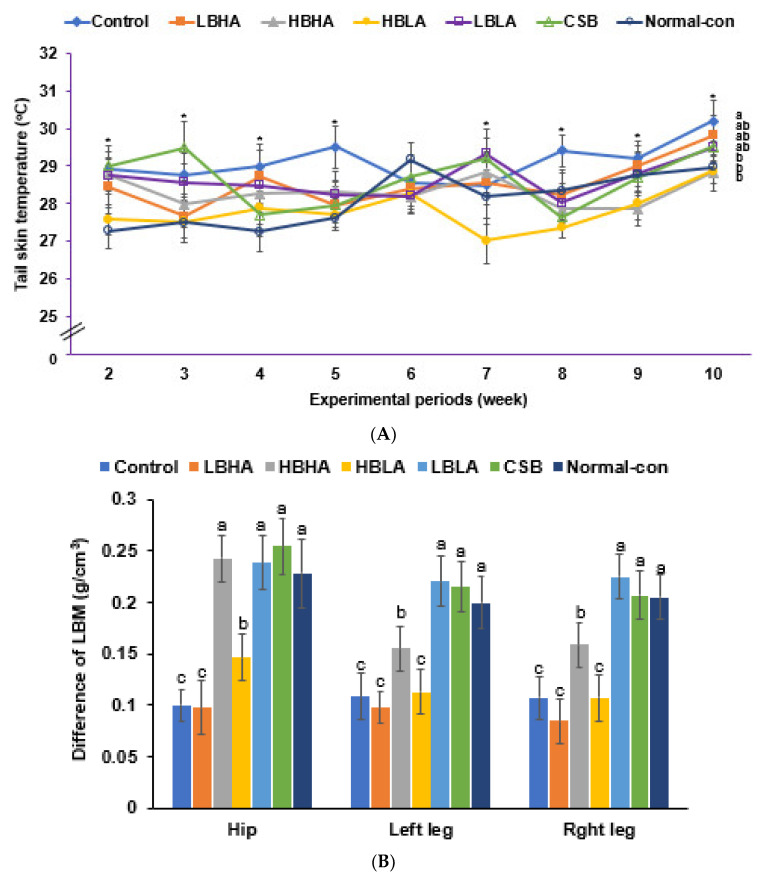

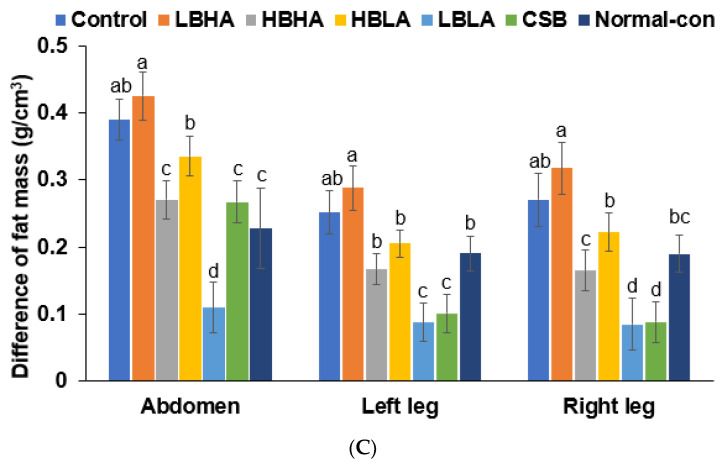
Tail skin temperature and skeletal muscle and fat mass. (**A**) Tail skin temperature during the experimental periods. (**B**) The difference in fat mass in the abdomen and leg fat mass before and after the intervention. (**C**) The difference in lean body mass (LBM) in the hip and leg before and after the intervention. The tail skin temperature represented a hot flush indication as the menopausal symptom was measured during the sleep cycle using an infrared thermometer for small rodents, three times and 10 min apart weekly. Since skeletal muscle and fat masses were changed after menopause, those of the leg, abdomen, and hip were measured using the dual-energy X-ray absorptiometer instrument equipped with software for assessing the body composition of small animals before and after the 12-week intervention. The differences in the fat mass and LBM in the abdomen, hip, and leg were calculated before and after the intervention. After menopause, skeletal muscle and fat mass decreased, and their improvement by TMD intake was shown. Dots and error bars represent the means ± standard deviations (*n* = 10). * Significant differences among the groups at *p* < 0.05. ^a,b,c,d^ Different letters on the bars indicate a significant difference among the groups by Tukey test at *p* < 0.05.

Due to ovariectomy, the uterine weight and serum 17β-estradiol concentrations were much lower in OVX compared to sham rats, and they was unaffected by TMD. The HBHA group showed a marginal increment in uterine weight and a non-significant increase in serum 17β-estradiol concentrations compared to the control (Table 2). Low estrogen in the OVX rats induced a higher tail skin temperature, and intake of TMD lowered it, and HBLA and HBHA intakes lowered the tail skin temperature to that of the normal control group (Figure 3A).

Body weight gain during the 12-week intervention was higher in the control than in the normal control group and was lower in the LBHA, HBHA, and HBLA groups than in the control group (Table 2). Food intake tended to be higher, but not significantly, in the control than the normal control group, whereas the TMD and CSB interventions did not alter the food intake. Food efficiency was much lower in the normal control than the control group, and the TMD and CSB interventions did not affect it (Table 2).

Uterine and retroperitoneal fat representing visceral fat mass (weight %) was higher in the control than the normal control group and was lower in the TMD groups compared to the control, except in the LBHA group—the visceral fat mass in the three TMD groups viz. HBHA, HBLA, and LBLA were similar to the normal control group (Table 2). DEXA revealed that the lean body mass (LBM) in the hips and legs of the LBHA group was similar to the control. It was lower than those of the HBHA, HBLA, and LBLA groups (Figure 3B). The fat mass in the abdomen and legs showed opposite results to that of the LBM in all the groups (Figure 3C). These results suggested that LBHA decreased the lean body mass (LBM) and increased the fat mass. However, its intake did not elevate weight gain and it could reduce the LBM.

### 3.4. Insulin Resistance and Lipid Profiles

Serum glucose concentrations in the fasting state were higher in the control group than in the normal control group. Fasting serum glucose concentrations of all TMD groups were at intermediate concentrations between the control and normal control groups, and all but the LBHA were significantly lower than the control group. At 2 h after food intake, serum glucose concentrations showed a similar trend to fasting serum glucose concentrations in all groups (Table 3). Fasting serum insulin concentrations were much higher in the control than in the normal control group and were lower in the HBHA, HBLA, and CSB groups. The concentrations in the HBLA and CSB groups were similar to the normal control group (Table 3). HOMA-IR, an indicator of insulin resistance, was much higher in the control than the normal control and the four TMD intake groups. The HOMA-IR in the HBLA and CSB groups was similar to the normal control group (Table 3).

The total cholesterol, HDL and LDL cholesterol, and triglyceride concentrations were elevated beyond the recommended range in the control compared to the normal control group. The TMD intake improved the lipid profiles compared to the control, and those in the HBHA, HBLA, and CSB groups were similar to the normal control group (Table 3). Serum LDL concentrations were lower in all TMD groups than not only the control but also the normal control group.

### 3.5. Lipid Metabolism in the Liver

Estrogen deficiency increased the serum glutamic oxaloacetic transaminase (GOT) and glutamic pyruvic transaminase (GPT) activities compared to the normal control group, and the TMD intake prevented the increase in OVX rats (Table 4). Hepatic glycogen storage decreased in the control group compared to the normal control group, and it was similar in the HBHA and HBLA groups to that seen in the normal control group (Table 4). Liver triglyceride and cholesterol contents were higher in the control than in the normal control group. TMD intake lowered the hepatic triglyceride content to the levels seen in the normal control group. However, the hepatic cholesterol content was lower in LBHA and LBLA groups, but not as low as in the normal control group (Table 4). The hepatic lipid contents were associated with the mRNA expressions of *PPAR-γ*, *SREBP-1c*, and *CPT-1*. *PPAR-γ* mRNA expressions related to hepatic insulin resistance were lower in the control than in the normal control group. The TMD intake increased its expression to as much as in the normal control group (Table 4). The *PPAR-γ* mRNA expression in the HBLA group was higher than in the normal control group. Hepatic *SREBP-1c* mRNA expression involved in cholesterol synthesis was higher in the control than in the normal control group, and its expression decreased in the LBHA, HBHA, HBLA, and CSB groups. The decrease in the expression of the hepatic *SREBP-1c* mRNA in the HBLA and the CSB groups was similar to that of the normal control group (Table 4). The hepatic *CPT-1* mRNA expression was lower in the control than in the normal control group, and an increase in expression was seen in HBHA, which is similar to its expression in the normal control group (Table 4).

### 3.6. Histology of the Large Intestines

**Figure 4:**
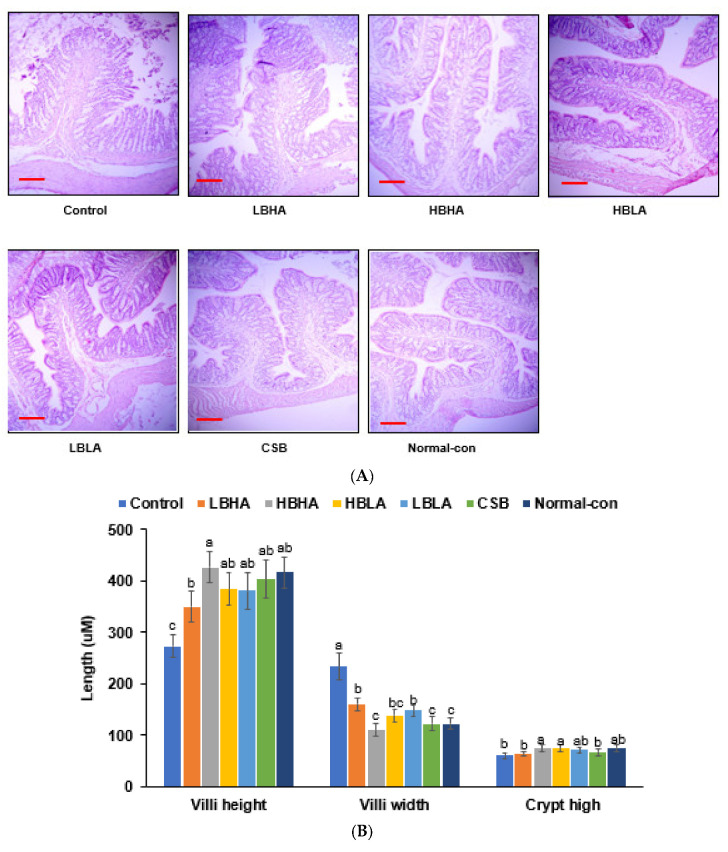

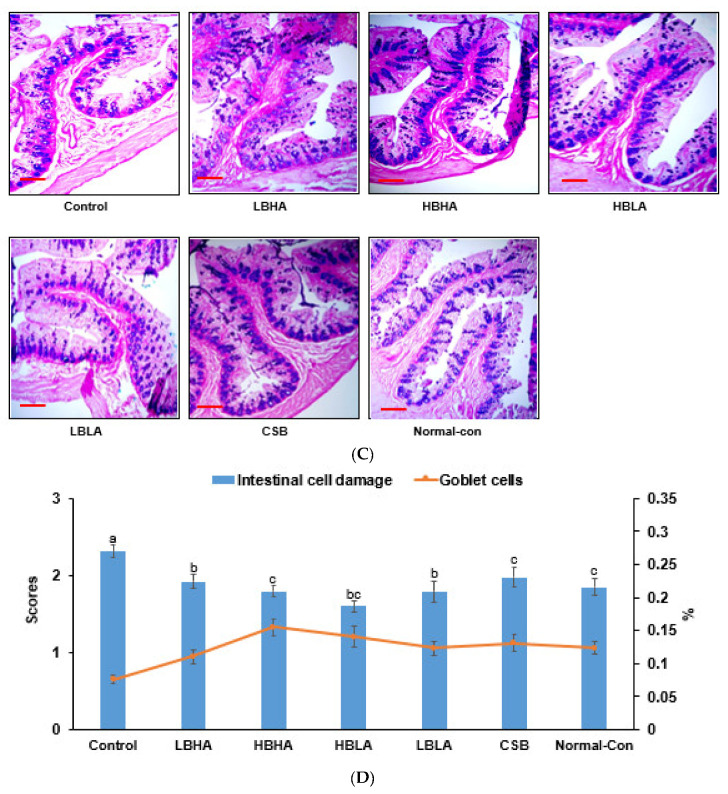
Intestinal morphology after the intervention. (**A**) Hematoxylin–eosin staining in the intestinal tissues. The red bar indicates the scale bar (10 um). (**B**) Intestinal villi length and width and crypt length. (**C**) Alcian blue–periodic acid–Schiff (PAS) stain in the intestinal tissues. The red bar indicates the scale bar (10 um), and the violet color indicates mucin. (**D**) Mucin and goblet cell contents. The paraffin-embedded large intestine was dissected into 10 um and was stained with H-E to show the intestinal morphology and Alcian blue–PAS to measure mucin contents. Since menopause could alter intestinal morphology and gut microbiota to modulate the host’s inflammation, energy, and glucose metabolism, the intestinal morphology was determined. Bars and error bars represent the means ± standard deviations (*n* = 10). ^a,b,c^ Different letters on the bars indicate a significant difference among the groups by Tukey test at *p* < 0.05.

The height of the villi of the large intestines was shortened in the control group compared to the normal control, and the TMD intake prevented the decrease. However, the increase in the villus height in the LBHA intake group was not as much as that seen in the normal control group (Figure 4A,B). The changes in the villi width were opposite to those in height. The villi width was much higher in the control than in the other groups, with HBHA being the lowest (Figure 4A,B). The crypt of the intestines was also smaller in the control group compared to normal controls, which is similar to the HBHA, HBLA, and LBLA groups (Figure 4A,B). The crypt in the CSB group was not significantly different from the control (Figure 4A).

The control group had much fewer mucin-producing goblet cells than the normal control group (Figure 4C,D). Interventions with TMD increased the number of mucin-producing goblet cells similar to that of the normal control group. HBHA and HBLA increased the number more than the normal control group (Figure 4C,D). The cell damage in the large intestinal tissues was also more severe in the control than in the normal controls, and the TMD interventions prevented the damage and were similar to the normal control group (Figure 4D). However, cell damage in the CSB was more severe than that seen in the normal control group.

### 3.7. SCFA in the Portal Vein and Gut Microbiota

**Figure 5:**
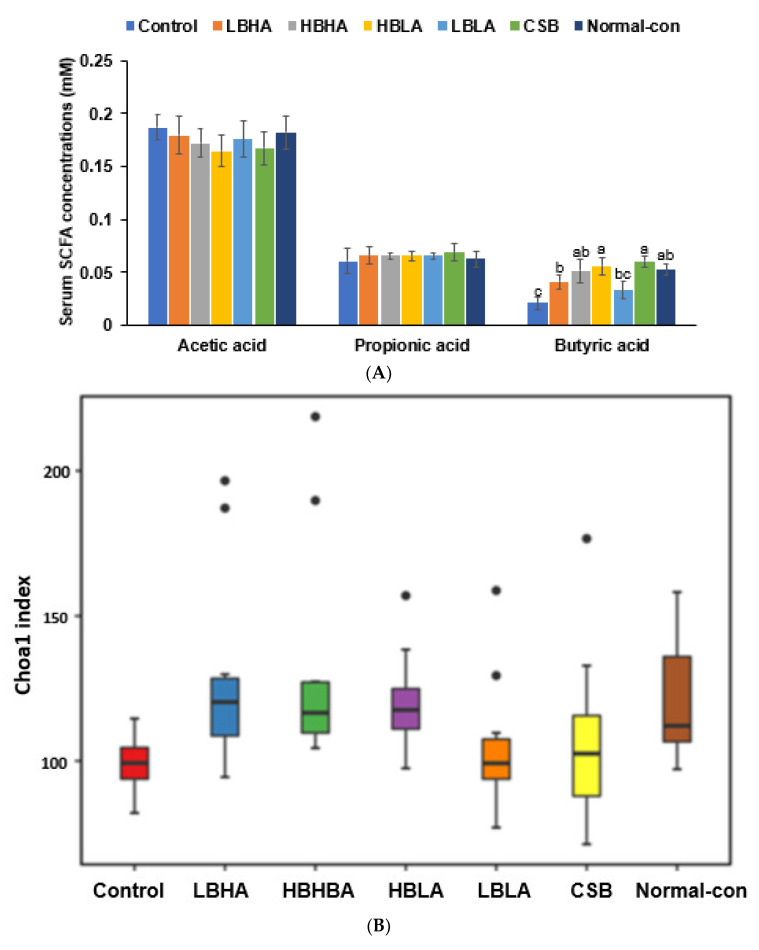

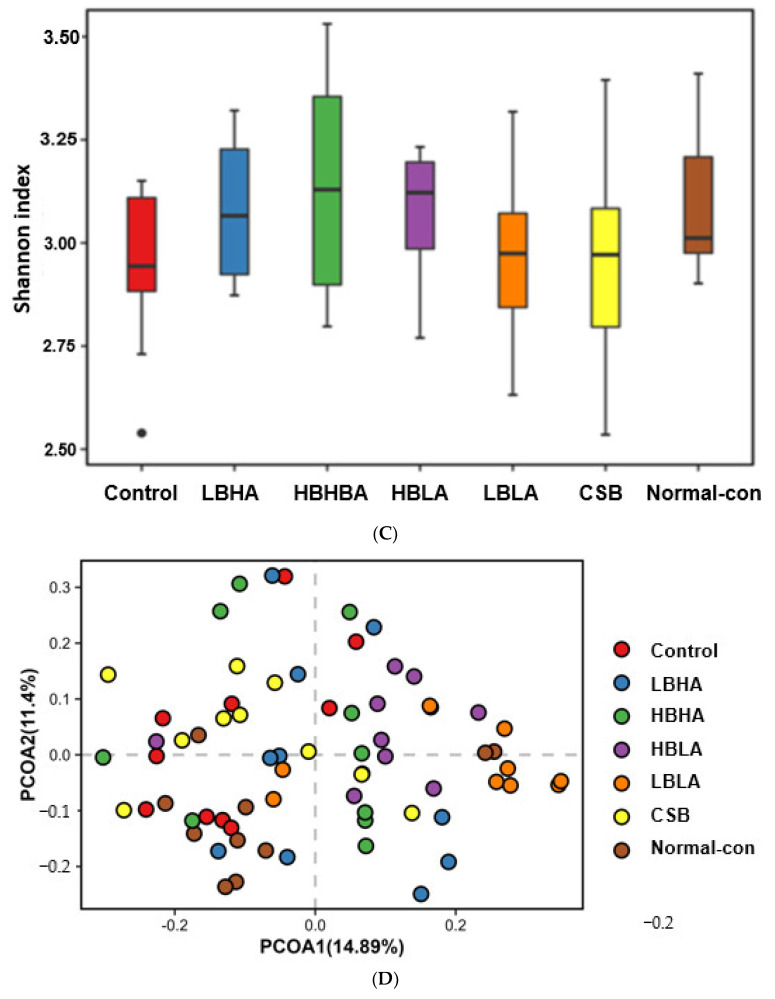

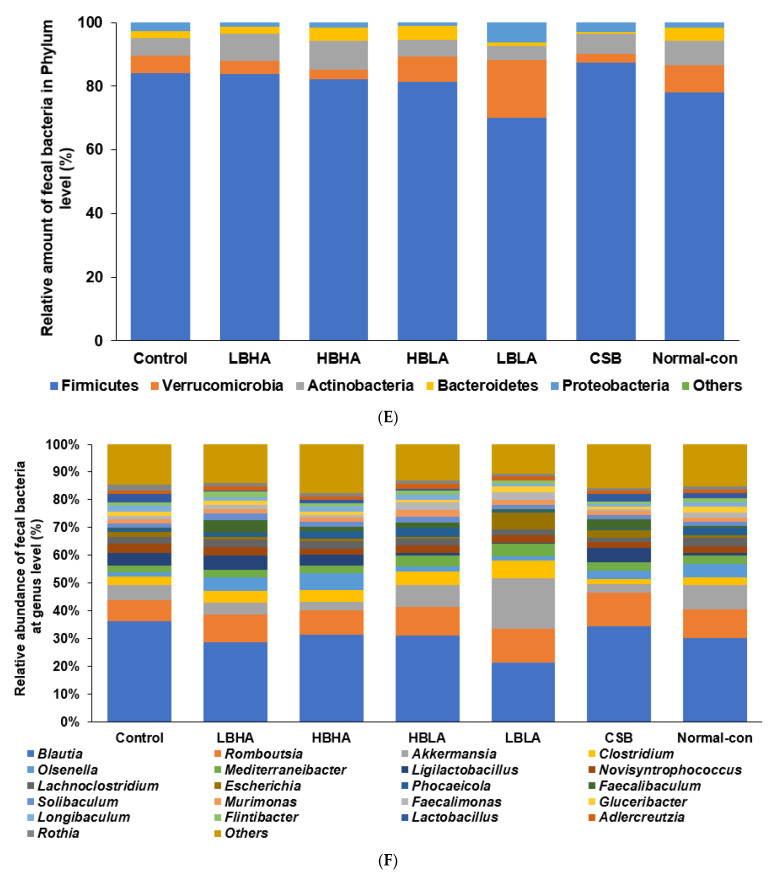
Short-chain fatty acid (SCFA) concentrations and cecal bacteria composition at the end of 12-week intervention. (**A**) SCFA concentrations in the blood from the portal vein. (**B**) α-diversity by Chao1 index in cecal bacteria. (**C**) α-diversity by Shannon index in cecal bacteria. (**D**) β-diversity in cecal bacteria. (**E**) Relative abundance of fecal bacteria from the cecum at the family level of cecal bacteria. (**F**) Relative abundance of fecal bacteria from the cecum at the genus level of cecal bacteria. SCFA (acetic, propionic, and butyric acids) contents in the serum from the portal vein were measured with gas chromatography. After the 12-week intervention, the bacteria in the collected cecal feces were measured with the NGS method, and their composition was analyzed with the Qiime2 program. Bacteria less than 1% were summed and shown as others. Menopause can alter gut microbiota and result in SCFAs in the portal vein, which influences the host metabolism, and TMD intake could prevent the alteration. We determined cecal bacteria composition and serum SCFA concentrations at the end of experimental periods. Bars and error bars represent the means ± standard deviations (*n* = 10). ^a,b,c^ Different letters on the bars indicate a significant difference among the groups by Tukey test at *p* < 0.05.

Portal vein acetate concentrations seemed to be lower in HBLA and CSB than in the control (*p* = 0.07), but there were no significant differences across all the groups (Figure 5A). Propionate concentrations did not differ significantly across all the groups. However, the butyrate concentrations were much lower in the control than the normal control, and they were highest in the HBLA and CSB groups and lowest in the LBHA and LBLA groups (Figure 5A).

The alpha diversity, determined by the Chao1 and Shannon indexes, was lower in the control compared to the normal control group, and the LBHA, HBHA, and HBLA interventions prevented their decrease (Figure 5B,C). However, LBLA and CSB intakes did not improve the α-diversity. A study of the β-diversity showed that the bacteria in the control and normal control groups and those in the TMD groups were distinct and separate (Figure 5D).

At the phylum level, the relative abundance of *Firmicutes* was higher in the control than the normal control and LBLA groups (Figure 5E). The relative abundance of *Verrucimicrobia* was higher in the LBLA and normal control groups than in the other groups, but that of *Proteobacteria* was also higher in the LBLA than other groups. At the genus level, the relative abundance of *Blutia* was higher, but *Romboutsia* was lower in the control compared to the other groups. The relative abundance of *Akkermentia* was higher in the LBLA and normal control groups than in the control (Figure 5F). The bacterial composition in the LBLA group was quite different from those of the other groups. The relative abundance of *Clostridium* and *Escherichia* was also higher in the LBLA than in the other groups (Figure 5F). At the genus level, the relative abundance of bacteria in the HBLA and HBHA was similar to that in the normal control group. CSB altered the gut microbiota composition as much as TMD (Figure 5F).

### 3.8. Metagenome Analysis of Fecal Bacteria

**Figure 6:**
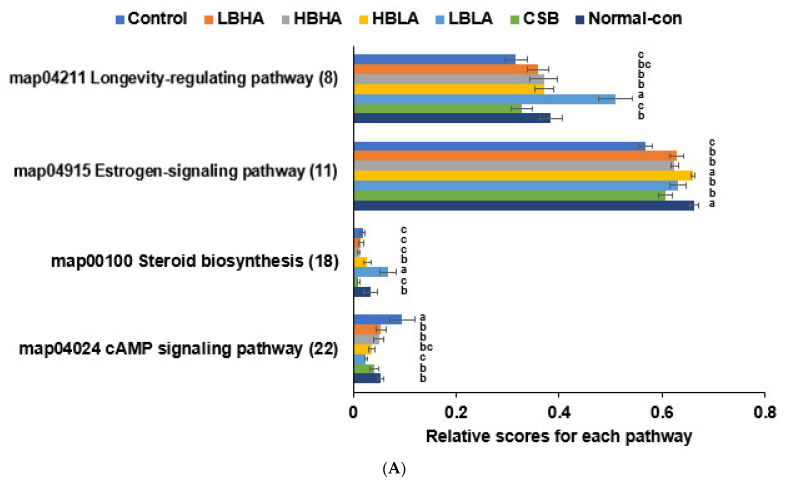

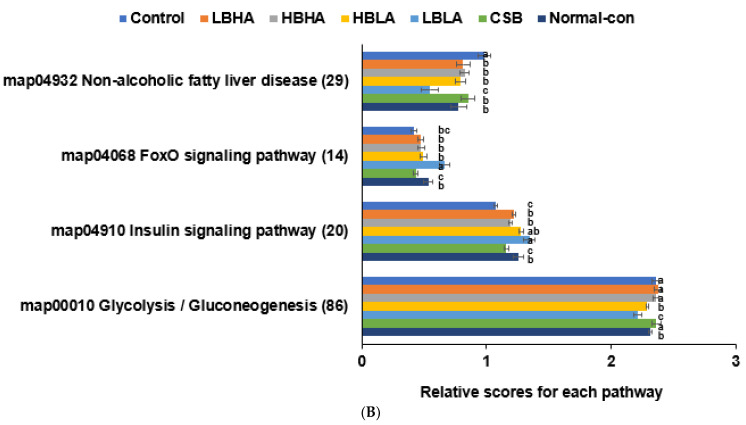
Metagenome analysis of cecal bacteria. (**A**) Estrogen signaling, steroid biosynthesis, and cAMP-signaling pathway. (**B**) Insulin-related signaling pathways. The changed composition of gut microbiota could alter their functions to influence the host-metabolism. The metagenome function of the cecal bacteria was determined with the Picrust2 program. Bars and error bars represent the means ± standard deviations (*n* = 10). ^a,b,c^ Different letters on the bars indicate a significant difference among the groups by Tukey test at *p* < 0.05.

The estrogen-signaling pathway was much more suppressed in the controls than in the normal controls. TMD and CSB increased it, and HBLA elevated it the most in the metagenome analysis of cecal bacteria by Picrust2 (Figure 6A). Steroid biosynthesis was also lower in the control than in the normal control. It increased with the HBLA and LBLA groups (Figure 6A). However, the cAMP-signaling pathway showed the opposite trend as the estrogen-signaling pathways (Figure 6A). The longevity-regulating pathway was lower in the control than the normal control and TMD, especially LBLA, tended to increase (Figure 6A).

The insulin-signaling pathway and its related pathway (FoxO signaling) were lower in the control than the normal control. They increased in TMD groups, especially HBLA and LBLA (Figure 6B). However, the glycolysis and gluconeogenesis pathway to increase glucose production was higher in the control than in the normal control and decreased in the LBLA and HBLA groups. A non-alcoholic fatty liver disease involved in hepatic insulin resistance was also higher in the control than the normal control and decreased in the TMD groups, especially LBLA (Figure 6B). These results suggested that gut microbiota in the HBLA and HBHA groups were linked to hepatic lipid metabolism.

## 4. Discussion

TMD and traditionally made kanjang comprise the soybean solids and liquid components of fermented soybeans in salty water, respectively. While the effects of TMD supplementation on obesity and blood glucose have been well-researched in previous animal and human studies [24,25,26], the presence of various bacteria and bioactive compounds in TMD may result in varying efficacies with different TMD samples. As seen in earlier studies, TMD primarily contains several beneficial bacteria, including *Bacillus, Lactobacillus, Pediococcus*, and *Weissella* spp. [14]. However, some varieties of TMD contain small amounts of unhealthy bacteria, such as *Enterobacter sakazakii*, *Acinetobacter baumannii*, and *Proteus mirabilis*, and compounds, such as biogenic amines [14]. *Acinetobacter baumannii* is an opportunistic nosocomial pathogen with multi-drug resistance, biofilm formation, and motility, and it can infect the host [27,28]. *Proteus mirabilis* also has similar activities to *Acinetobacter baumannii* in animals [29]. *Enterobacter sakazakii* is also an opportunistic foodborne pathogen that can induce necrotizing enterocolitis, bacteremia, and meningitis [30]. However, the amounts of *Enterobacter sakazakii*, *Acinetobacter baumannii*, and *Proteus mirabilis* were small, and the animals did not show infectious disease symptoms in any groups in the present study. Therefore, TMD, regardless of Bacillus and biogenic amine contents, was shown to be a safe food, and TMD with high *Bacillus* spp. alleviated estrogen-deficient symptoms in OVX rats. However, the results need to be confirmed in a clinical study.

Cooked soybeans include isoflavonoid glycones and aglycones, and their fermentation with *Bacillus* spp. was shown to change the isoflavonoid glycones to isoflavonoid aglycones in a previous study [31]. In the present study, LBHA, HBHA, and HBLA did not contain isoflavonoid glycones and they had increased amounts of isoflavonoid aglycones compared to CSB. However, LBLA contained isoflavonoid glycones, indicating that fermentation was not sufficient. Soybeans fermented for short periods, such as chungkookjang made by traditional methods or by fermentation with *Bacillus amyloliquefaciens*, have decreased isoflavonoid glycones and increased isoflavonoid aglycones. Isoflavonoid aglycones have a better efficacy than isoflavonoid glycones in human intestines [32], and specifically, daidzein can be potentially converted into equol with potent estrogenic activity [9]. Therefore, the predominant bacillus species and the duration of fermentation determine the conversion of isoflavonoid glycones into isoflavonoid aglycones.

Some TMDs contain biogenic amines produced by amino acid decarboxylation by bacteria. The biogenic amines include tryptamine, 2-phenyl–ethylamine, putrescine, cadaverine, agmatine, histamine, tyramine, spermidine, and spermine, ranging within 18–245 mg%. The primary ones are histamine and tyramine in doenjang [33]. Biogenic amines can be toxic, and they need to be controlled. The present study demonstrated that TMD with high biogenic amines contained about 109–165 mg% for tyramine and 50–63 mg% for histamine, and their intake did not show harmful effects on estrogen deficiency symptoms in an animal model. Previous studies have demonstrated the reduction in biogenic amines in fermented soybean foods [14,34], but their contents in TMD might not be detrimental to the metabolism. The biogenic amine contents might be influenced by fermentation conditions such as temperature, sodium contents, bacterium types, fermentation periods, and others. Previous studies have demonstrated that the Bacillus spp., especially *Bacillus licheniformis*, degrades biogenic amines [35,36]. The present study showed that HBLA mainly contained Bacillus spp., but HBHA included high Bacillus spp. and other bacteria (*Leuconostoc mesenteroides*, *Pediococcus acidilactici*, and *Weissella confusa*) as well. The results suggested that TMD containing mostly Bacillus spp. might be lower in biogenic amines.

Estrogen deficiency leads to peripheral vasodilation, which causes hot flashes and excessive sweating in the face, neck, and chest [37]. A hot flash results from elevated central sympathetic activation through the α2-adrenergic, serotonergic, and dopaminergic receptors and is ameliorated by modulating its activation [38,39]. A high-fat diet and obesity may exacerbate menopausal symptoms, including hot flashes [40,41]. The present study used high-fat diets to exacerbate menopausal symptoms. Hot flashes measured by the tail skin temperature were elevated in OVX rats above the temperatures in sham-operated rats, whereas OVX rats fed HBHA and HBLA showed decreased tail skin temperatures, which was similar to the sham rats. However, OVX rats fed CSB and TMD exhibited intermediate tail skin temperatures between the control and normal control groups. Consistent with the present study, soy isoflavonoids have been shown to attenuate hot flashes in menopausal women [42,43]. Furthermore, previous studies have demonstrated that estrogen injections can alleviate hot flashes and reduce the selective serotonin reuptake inhibitor, selective serotonin-norepinephrine reuptake inhibitor, gamma-aminobutyric acid analog, and α-adrenergic receptor agonist [39]. Isoflavonoid aglycones, such as daidzein and genistein, act as partial selective estrogen receptor modulators for improving menopausal symptoms [44].

Estrogen acts as a regulator of energy metabolism, including energy intake and expenditure [45]. Post-menopause decreases energy expenditure and is consistently reported to be linked to decreased skeletal muscle mass [46]. It is related to decreased skeletal muscle mass and muscle dysfunction, which is caused by a decreased proliferation of muscle satellite cells and increased levels of inflammatory markers [47]. The present study showed that the estrogen-deficient (control group) rats exhibited an increased visceral fat and decreased lean body mass compared to those in the normal control group. TMD, especially HBHA and HBLA interventions, decreased the visceral fat mass and increased the lean body mass without increasing the serum 17β-estrogen concentrations. Tang et al. also demonstrated that soy foods prevent obesity and osteosarcopenia [48]. However, in the metagenome analysis of cecal microbiota, the estrogen-signaling pathways and steroid biosynthesis were elevated in the HBHA, HBLA, and LBLA groups compared to the control. Therefore, TMD interventions may improve energy metabolism via the gut metagenome.

Menopause disrupts lipid metabolism, which causes serum dyslipidemia and fat deposits in the liver [49]. Consistent with previous research [48,49], our results also show a deterioration of lipid metabolism by elevating cholesterol and triglyceride biosynthesis and decreasing lipid utilization in the liver in OVX rats. HBHA and HBLA prevented the disturbance of hepatic lipid metabolism, which could be linked to increased isoflavonoid aglycones and *Bacillus subtitles* in HBHA and HBLA. Soybean intake is one of the alternative therapies that is often recommended to improve menopausal symptoms and normalize energy, glucose, and lipid metabolism [48]. The isoflavonoids, oils, and proteins in soybeans are reported to decrease liver fat deposition by promoting adiponectin-mediated AMP-activated protein kinase-α pathways in rats that are fed high-fat and -cholesterol diets [50,51]. The present study also showed that CSB improved hepatic lipid metabolism compared to the control group, and HBHA and HBLA showed slightly better improvement in hepatic lipid metabolism than CSB. Furthermore, the TMD intake, especially HBHA and HBLA, was more effective in reducing the hepatic fat deposition by decreasing the fatty acid synthase activity and increasing the CPT-1 activity in the liver than unfermented soybeans, as shown in a previous study [52].

The gut microbiota influences various metabolic activities in the host. Menopause is associated with lower gut microbiota diversity, and there is a shift of the gut microbiota in post-menopausal women toward that observed in men [53]. It indicates that the action of estrogen could be linked with the gut microbiota, although this involvement remains inconsistent. Since the changes in the bioactive components in soybeans are also associated with the host’s gut microbiota, the effects of the soybean intake on the host metabolism are somewhat varied [7]. Long-term soybean consumption can modulate the gut microbiota to improve its effectiveness. Soybean intake is generally reported to increase the ratio of *Firmicutes* and *Bacteoidetes*, *Bifidobacterium*, and *Lactobacilli* and to decrease pathogenic bacteria [54]. This study also demonstrates that control rats had a lower α-diversity, and LBHA, HBHA, and HBLA prevented a further decrease in diversity. However, the impact on microbiota diversity with the intake of LBLA and CSB was lower compared to other TMDs. The intake of fermented soybeans has been reported to alter the gut microbiota to shift to *Lactobacillus* and *Bifidobacterium* as the predominant genera. However, the TMD intake has been shown to result in a decrease in the *Firmicutes* to *Bacteroidetes* ratio in the gut microbiota and a significant decrease in the abundance of *Ruminococcaceae* and *Lachnospiraceae* while that of *Odoribacter* increased [55]. We also showed changes similar to the study mentioned above, and *Akkermentia* increased in the LBLA and HBLA groups, which was similar to the normal control. Therefore, the improvements in energy and lipid metabolism seen with TMD intake, especially HBHA, and HBLA, might be linked to improvements in the hepatic fat metabolism, stimulation of the estrogen and insulin-signaling pathways by the gut microbiome, and increases in isoflavonoid aglycones.

The limitations of the present study were that the (1) TMD was categorized into four types according to the contents of the beneficial bacteria and biogenic amine contents. However, some could not belong to the categories. (2) The reproducibility of the TMD could not be high in later studies, although we checked over five different batches, demonstrating the low variability of bacteria and biogenic amines in the same product. Despite the limitations, this study could give insight into alleviating menopausal symptoms by consuming TMD with high *Bacillus* spp. and *Bacillus* spp. that are rich in HBLA and HBHA, which could be developed as a starter for standardized doenjang in the future.

In summary, HBLA and HBHA, among the TMD varieties, which were produced by the long-term fermentation of soybeans with salt, contained higher levels of isoflavonoid aglycones and *Bacillus subtilus*, but no opportunistic bacteria. In particular, HBLA contained mostly *Bacillus subtilus*, which increased isoflavonoid aglycones and low biogenic amines. The intake of HBLA and HBHA, including an abundance of *Bacillus* spp., ameliorated hot flashes and decreased the visceral fat mass and hepatic lipid deposition via the stimulation of the *PPAR-γ* and *CPT-1* mRNA expressions. The improvement was marginally better than that of CBS but not significantly different. Moreover, the intake of TMD, especially HBLA, enhanced estrogen and insulin signaling, decreased cAMP-signaling pathways in the cecal microbiota, and improved intestinal morphology better than CSB. In conclusion, the long-term intake of HBLA and HBHA reduced hot flashes and restored energy and lipid metabolism homeostasis induced by estrogen deficiency, potentially better than CSB, via the modulating gut microbiota. TMD containing high levels of *Bacillus* spp. and isoflavonoid aglycones (HBLA and HBHA) can be used daily during cooking as a substitution for salt to prevent and ameliorate estrogen deficiency symptoms in women. Furthermore, *Bacillus subtilus* can be used as a starter for standardized doenjang, but it needs more research to demonstrate that it can produce a high-quality doenjang.

## Figures and Tables

**Table 1:** Isoflavonoids biogenic amine, and sodium contents of each traditionally made doenjang (TMD).

Groups	LBHA	HBHA	HBLA	LBLA
Total isoflavonoid aglycones (mg/kg)	0.54 ± 0.06 ^b^	0.50 ± 0.02 ^b^	0.67 ± 0.00 ^a^	0.28 ± 0.05 ^c^
Daidzein (mg/kg)	0.21 ± 0.03 ^b^	0.22 ± 0.01 ^b^	0.26 ± 0.00 ^a^	0.09 ± 0.02 ^c^
Genistein (mg/kg)	0.28 ± 0.03 ^b^	0.25 ± 0.01 ^b^	0.36 ± 0.00 ^a^	0.12 ± 0.03 ^c^
Glycitein (mg/kg)	0.05 ± 0.01 ^a^	0.04 ± 0.00 ^a^	0.04 ± 0.00 ^a^	0.01 ± 0.00 ^b^
Daidzin (mg/kg)	-	-	-	0.02 ± 0.01
Genistin (mg/kg)	-	-	-	0.03 ± 0.01
Glycitin (mg/kg)	-	-	-	-
Histamine (mg/kg)	501 ± 0.78 ^b^	628 ± 0.34 ^a^	13.1 ± 0.10 ^d^	33.2 ± 0.20 ^c^
Tyramine (mg/kg)	1655 ± 0.81 ^a^	1088 ± 0.80 ^b^	20.8 ± 0.73 ^d^	155 ± 0.83 ^c^
Sodium (wt %)	5.14 ± 0.04 ^a^	4.37 ± 0.10 ^b^	4.87 ± 0.04 ^a^	4.52 ± 0.08 ^ab^

Values represented means ± standard deviation (*n* = 5). HBLA, TMD with high contents of *Bacillus* spp. and high biogenic amines. HBHA, TMD with high contents of *Bacillus* spp. and low biogenic amines. LBHA, TMD with low contents of *Bacillus* spp. and high biogenic amines. LBLA, TMD with low contents of *Bacillus* spp. and low biogenic amines. ^a,b,c,d^ Different letters on the bars indicate a significant difference among the groups by Tukey test at *p* < 0.05.

**Table 2:** Energy metabolism at the end of the experiment.

	Control	LBHA	HBHA	HBLA	LBLA	CSB	Normal Con
Uterine weight (g)	0.15 ± 0.02 ^c^	0.14 ± 0.02 ^c^	0.21 ± 0.03 ^b^	0.13 ± 0.02 ^c^	0.12 ± 0.02 ^c^	0.15 ± 0.03 ^c^	0.65 ± 0.03 ^a^
Serum 17β-estradiol (pg/mL)	1.44 ± 0.11 ^b^	1.38 ± 0.13 ^b^	1.59 ± 0.22 ^b^	1.37 ± 0.21 ^b^	1.35 ± 0.26 ^b^	1.49 ± 0.18 ^b^	6.36 ± 0.85 ^a^
Final weight (g)	351 ± 26.7 ^a^	324 ± 17.9 ^b^	330 ± 18.6 ^b^	321 ± 24.9 ^b^	319 ± 28.9 ^b^	326 ± 31.5 ^b^	277 ± 28.6 ^c^
Weight gain (g)	172 ± 12.7 ^a^	148 ± 12.8 ^b^	159 ± 10.3 ^b^	157 ± 16.5 ^b^	164 ± 17.9 ^ab^	164 ± 18.6 ^ab^	113 ± 21.9 ^c^
Food intake (g/day)	13.4 ± 1.7	11.8 ± 1.3	12.3 ± 0.7	12.3 ± 1.3	13.6 ± 1.3	12.7 ± 1.2	11.4 ± 1.3
Food efficiency	13.4 ± 1.6 ^a^	12.6 ± 1.3 ^a^	13.1 ± 0.7 ^a^	12.7 ± 1.3 ^a^	12.2 ± 1.1 ^a^	12.9 ± 1.4 ^a^	9.8 ± 1.4 ^b^
Uterine fat (g)	10.3 ± 0.92 ^a^	10.4 ± 1.14 ^a^	7.86 ± 0.97 ^b^	7.58 ± 0.87 ^b^	5.73 ± 0.9 ^c^	7.08 ± 0.87 ^b^	6.04 ± 0.97 ^c^
Retroperitoneal fat (g)	5.02 ± 0.63 ^a^	4.46 ± 0.53 ^a^	3.65 ± 0.37 ^b^	3.55 ± 0.44 ^b^	2.55 ± 0.37 ^c^	2.88 ± 0.32 ^c^	3.94 ± 0.98 ^b^
Visceral fat (% of bw)	4.72 ± 0.35 ^a^	4.98 ± 0.44 ^a^	4.10 ± 0.42 ^b^	4.09 ± 0.42 ^b^	3.03 ± 0.41 ^c^	3.76 ± 0.38 ^b^	3.93 ± 0.67 ^b^

Values represent the means ± standard deviations (*n* = 10). bw, body weight. HBLA, traditionally made doenjang (TMD) with high contents of *Bacillus* spp. and high biogenic amines. HBHA, TMD with high contents of *Bacillus* spp. and low biogenic amines. LBHA, TMD with low contents of *Bacillus* spp. and high biogenic amines. LBLA, TMD with low contents of *Bacillus* spp. and low biogenic amines. ^a,b,c^ Different letters on the bars indicate a significant difference among the groups by Tukey test at *p* < 0.05.

**Table 3:** Serum glucose and lipid profiles at the end of the experiment.

	Control	LBHA	HBHA	HBLA	LBLA	CSB	Normal Con
Fasting serum glucose (mg/dL)	116 ± 4.15 ^a^	108 ± 6.81 ^ab^	106 ± 5.95 ^b^	105 ± 8.02 ^b^	111 ± 7.06 ^ab^	111 ± 8.68 ^ab^	98.2 ± 7.08 ^c^
2 h post-prandial serum glucose (mg/dL)	148 ± 7.84 ^a^	138 ± 7.52 ^ab^	129 ± 7.1 ^b^	129 ± 9.53 ^b^	143 ± 7.91 ^a^	136 ± 6.38 ^ab^	129 ± 6.79 ^b^
Fasting serum insulin (ng/mL)	1.52 ± 0.17 ^a^	1.47 ± 0.17 ^a^	1.07 ± 0.11 ^b^	0.80 ± 0.17 ^c^	1.34 ± 0.17 ^ab^	0.89 ± 0.17 ^c^	0.89 ± 0.09 ^c^
HOMA-IR	6.27 ± 0.09 ^a^	6.39 ± 0.05 ^a^	4.26 ± 0.06 ^b^	3.26 ± 0.09 ^c^	4.85 ± 0.06 ^b^	3.05 ± 0.1 ^c^	3.94 ± 0.98 ^bc^
Serum total cholesterol (mg/dL)	209 ± 23.4 ^a^	183 ± 18.3 ^ab^	154 ± 14.0 ^b^	154 ± 11.3 ^b^	164 ± 17.0 ^b^	155 ± 23.3 ^b^	200 ± 21.9 ^a^
Serum HDL (mg/dL)	43.7 ± 3.71 ^c^	53.4 ± 4.95 ^b^	55.6 ± 4.08 ^ab^	61 ± 3.17 ^a^	47 ± 4.8 ^c^	54.1 ± 4.12 ^ab^	59.6 ± 1.92 ^a^
Serum LDL (mg/dL)	142 ± 14.6 ^a^	116 ± 10.3 ^b^	83.8 ± 7.89 ^d^	80.9 ± 5.36 ^d^	99.3 ± 10.3 ^c^	88.8 ± 11.8 ^d^	127 ± 9.92 ^b^
Serum TG (mg/dL)	115 ± 9.59 ^a^	71.8 ± 12.8 ^c^	74.4 ± 4.61 ^c^	57.9 ± 6.04 ^d^	87.8 ± 9.9 ^b^	57.9 ± 6.05 ^d^	66.6 ± 9.92 ^cd^

Values represent the means ± standard deviations (*n* = 10). HOMA-IR, homeostatic model assessment for insulin resistance; HDL, high-density lipoprotein; LDL, low-density lipoprotein; TG, triglyceride. HBLA, traditionally made doenjang (TMD) with high contents of *Bacillus* spp. and high biogenic amines. HBHA, TMD with high contents of *Bacillus* spp. and low biogenic amines. LBHA, TMD with low contents of *Bacillus* spp. and high biogenic amines. LBLA, TMD with low contents of *Bacillus* spp. and low biogenic amines. ^a,b,c,d^ Different letters on the bars indicate a significant difference among the groups by Tukey test at *p* < 0.05.

**Table 4:** Liver damage and hepatic glucose and lipid metabolism.

	Control	LBHA	HBHA	HBLA	LBLA	CSB	Normal Con
Serum AST (IU/L)	63.5 ± 4.16 ^a^	49.4 ± 3.71 ^c^	56.6 ± 2.61 ^b^	54.3 ± 3.57 ^b^	54.2 ± 3.22 ^b^	55.6 ± 2.48 ^b^	56.1 ± 2.27 ^b^
Serum ALT (IU/L)	32.8 ± 3.86 ^a^	28 ± 4.26 ^b^	16.1 ± 2.84 ^c^	18.3 ± 3.46 ^c^	29.6 ± 4.39 ^ab^	16.9 ± 2.44 ^c^	17.9 ± 1.63 ^c^
Glycogen (mg/g tissue)	24.4 ± 3.45 ^b^	29.1 ± 4.27 ^ab^	34.5 ± 3.75 ^a^	35.5 ± 2.79 ^a^	27.7 ± 3.63 ^b^	28.3 ± 3.94 ^b^	31.7 ± 4.05 ^a^
TG (mg/g tissue)	294 ± 11.8 ^a^	223 ± 15.2 ^c^	218 ± 12.9 ^c^	229 ± 18.6 ^c^	241 ± 11.2 ^b^	252 ± 16.4 ^b^	243 ± 12.1 ^b^
Cholesterol (mg/g tissue)	369 ± 10.7 ^a^	291 ± 13.0 ^c^	367 ± 18.0 ^a^	367 ± 16.8 ^a^	332 ± 13.5 ^b^	385 ± 9.60 ^a^	255 ± 14.5 ^d^
*PPAR-γ* mRNA (AU)	1 ± 0 ^c^	2.69 ± 0.47 ^b^	2.67 ± 0.43 ^b^	3.27 ± 0.33 ^a^	2.91 ± 0.29 ^b^	2.8 ± 0.42 ^b^	2.57 ± 0.29 ^b^
*SREBP-1c* mRNA (AU)	1 ± 0 ^a^	0.81 ± 0.06 ^b^	0.65 ± 0.11 ^bc^	0.47 ± 0.08 ^c^	0.94 ± 0.13 ^a^	0.41 ± 0.04 ^c^	0.42 ± 0.06 ^c^
*CPT-1* mRNA (AU)	1 ± 0 ^c^	1.52 ± 0.16 ^b^	2.01 ± 0.19 ^a^	1.56 ± 0.17 ^b^	1.2 ± 0.13 ^c^	1.59 ± 0.19 ^b^	2.02 ± 0.31 ^a^

Values represent the means ± standard deviations (*n* = 10). AST, aspartate aminotransferase; ALT, alanine aminotransferase; TG, triglyceride; *PPAR-γ*, peroxisome proliferator-activated receptor-γ; *SREBP-1c*, sterol regulatory element-binding protein-1c; *CPT-1*, carnitine palmitoyltransferase-1. HBLA, traditionally made doenjang (TMD) with high contents of *Bacillus* spp. and high biogenic amines. HBHA, TMD with high contents of *Bacillus* spp. and low biogenic amines. LBHA, TMD with low contents of *Bacillus* spp. and high biogenic amines. LBLA, TMD with low contents of *Bacillus* spp. and low biogenic amines. ^a,b,c,d^ Different letters on the bars indicate a significant difference among the groups by Tukey test at *p* < 0.05.

## Data Availability

The data presented in this study are available on request from the corresponding author.
